# British Health Stations.—II

**Published:** 1904-04-23

**Authors:** 


### BRITISH HEALTH STATIONS.—II.

(By Our Special Medical Commissioner.)

(*Continued from page* 361.)

### THE ISLE OF WIGHT.

With the coming of Spring there awakens in most individuals a desire for rejuvenescence, a longing for re-creation of body, and re-adjustment of mind. In this climate the winter bears heavily on the worker, be he labourer with brain or muscle, and most find that with the lengthening of the days there comes a shortening of vital energy. The overdraft on life's bank is often heavy, and it is not easy always to recoup. The spring holiday is becoming an absolute necessity. Travel has long been reckoned as a luxury for the leisured and well-to-do, but the demands of the present-day life, at least as lived in busy centres with incessant and unavoidable stress and strain, make some form of vagabondage a prophylactic of the highest value.

In this country it is not altogether easy in the springtime to select a station which shall afford material for the invigoration of mind and opportunities for the re-establishment of body. One of the most suitable resorts for health and pleasure is undoubtedly the Isle of Wight. We have visited this far-famed "Garden of England" at all seasons of the year, but have no hesitation in giving the opinion that the springtime is by far the best period in which to study the charms and peculiar attractions of Vectis. This compact little island, separated from Hampshire by the Solent, is readily accessible, affords much variety, contains a number of very different and exceedingly desirable towns and hamlets with modern conveniences for delicate visitors, presents abundant opportunities for active outdoor life and sport for the vigorous, and for the student is rich in historical, archæological, geological, and botanical features. The climate is essentially marine and mild, and while generally equable, is breezy, and, except in certain sheltered districts, fairly bracing. Considerable variety, however, may be obtained, the invigorating Downs of Freshwater differing much from the sheltered undercliff.

The island has very rightly won distinction as an admirable convalescing station. For the invalid and debilitated, Ventnor, Bonchurch, Niton, or Blackgang, will probably be selected. The tired worker and wearied school-child will be braced by sojourn at Brading, Sandown, and Shanklin on the east, or by the more secluded life of Freshwater or Totland Bay at the western extremity. For the mere holiday-maker a roving commission may be granted, and much that is full of charm and health-bringing invigoration may be discovered by cycling along inland lanes and by-roads, or tramping along the chalky downs which run across the island. The Isle of Wight has long found favour with holiday-makers; it should be better known and more wisely used as one of the best of England's island sanatoria for service in the springtime.

An excellent service of trains from London and convenient steamboats for crossing the Solent make this particularly desirable spring resort comparatively easy of access.

Ample accommodation is available in almost all parts of the Island and sufficiently varied as to meet the requirements of all classes of cases.

We shall hope to deal in future articles with some of the different places in the Island which more particularly meet the needs of health-seekers.

